# Dr. Oscar Fitzerland Miller

**Published:** 1908-11

**Authors:** 


Dr. Oscar Fitzerland Miller, died at the home of his son at Flint, Mich., August 11, 1908, of heart disease, aged 77 years. He graduated at the University of Buffalo, Medical Department, in 1858, afterward serving as a medical officer during the Civil War.

